# Maternal diet and human milk composition: an updated systematic review

**DOI:** 10.3389/fnut.2023.1320560

**Published:** 2024-01-23

**Authors:** Inga Petersohn, Anneke H. Hellinga, Linde van Lee, Nicole Keukens, Louis Bont, Kasper A. Hettinga, Edith J. M. Feskens, Elske M. Brouwer-Brolsma

**Affiliations:** ^1^Division of Human Nutrition and Health, Wageningen University and Research, Wageningen, Netherlands; ^2^Ausnutria BV, Zwolle, Netherlands; ^3^Center for Translational Immunology, University Medical Center Utrecht, Utrecht, Netherlands; ^4^Department of Paediatric Immunology and Infectious Diseases, Wilhelmina Children’s Hospital, University Medical Center Utrecht, Utrecht, Netherlands; ^5^ReSViNET Foundation, Zeist, Netherlands; ^6^Division of Food Quality and Design, Wageningen University and Research, Wageningen, Netherlands

**Keywords:** maternal diet, breastfeeding, milk composition, infants, human milk

## Abstract

**Context:**

Exclusive breastfeeding for 6 months after birth provides infants with the best start for life. A review by Bravi et al. summarized the importance of maternal diet as a determinant of human milk composition based on data up to 2015, but evidence on nutrient intake level was limited.

**Objective:**

We updated the review by Bravi et al., critically assessed differences in study designs and sampling methods, and graphically visualized trends and associations.

**Data sources:**

PubMed was systematically searched for articles published between January 2015 and March 2021.

**Data extraction:**

Article screening, selection, and data extraction was done by two independent researchers, including a risk of bias assessment based on 11 criteria. Articles were eligible when including: quantitative information, commonly used effect estimates, healthy mother-infant dyads.

**Results:**

Twenty seven observational and five intervention studies were identified (*n* = 7,138) and combined with results of Bravi et al. Fatty acids were still the most studied human milk components in relation to maternal diet (*n* = 17 studies) with maternal fish intake being predominantly positively associated with milk ALA (*r* = 0.28–0.42), DHA (*r* = 0.24–0.46), and EPA (*r* = 0.25–0.28) content. PUFAs from diet were generally positively correlated with their concentrations in milk, while SFA intake was negatively associated with several fatty acids in milk. Studies on associations with maternal diet and milk carbohydrates, proteins, vitamins and minerals were limited in number and varied in methods and results.

**Conclusion:**

This updated review shows that evidence on the association between maternal diet and human milk fatty acids is rapidly increasing, but still diversified in methodology and results. Further studies, preferably intervention studies, assessing diet and milk carbohydrates, proteins, vitamins and minerals are needed to be able draw conclusions on the importance of maternal diet for human milk composition as a whole.

## Introduction

According to the WHO, exclusive breastfeeding during the first 6 months after birth provides the infant with the most optimal start for life (1). The WHO additionally states that the nutrient composition of human milk is essential for growth, development and health of infants and that breastfeeding is an unequaled way of feeding (1, 2). Accordingly, it has been suggested that breastfed infants are less prone to excess weight gain, diabetes, childhood infections and more prone to an increased intelligence (3, 4).

To unravel the exact underlying pathways of the associations between human milk and infant health, recent studies aimed to gain more insight in the role of specific nutrients that may explain the observed health benefits in breastfed children. However, studies on human milk nutrient composition and infant health are challenging as human milk composition is not constant and changes dynamically, e.g., influenced by time of the day, lactation duration, and time since last feeding (5). Various maternal and child related factors have been associated to human milk composition as well, such as maternal age, ethnicity, gestational weight gain, infant’s birth weight (5), gestational age at birth (6), genetics (7), maternal tobacco smoking (8), and maternal diet (9). Focusing on the latter, findings suggest that human milk fatty acids and various fat- and water-soluble vitamins are influenced by maternal dietary intake of those nutrients (10). Furthermore, differences in human milk nutrient composition between mothers with vegan, vegetarian or omnivore diets have been reported (11, 12).

A systematic review published in Bravi et al. (9) examined the associations between maternal diet and human milk composition. In their review, 36 papers were included and most of them studied influences of maternal diet on the human milk fatty acid profile. While there were some promising results showing significant positive association of maternal fish intake and human milk DHA and n-3 PUFA content, Bravi and colleagues concluded that evidence is scarce and outcome and exposure variables too diverse to draw strong conclusions. Studies on other nutrients were even more limited. Since 2015, a substantial number of new studies covering this topic have been published. Therefore, we aimed to perform an updated systematic review adding to Bravi and colleague’s previous work, to critically assess differences in study designs and sampling methods, and graphically visualize trends and associations.

## Methods

### Search strategy

A systematic PubMed search was carried out, limited to articles published between January 2015 and March 2021, based on the latest date included by Bravi et al. (9). The search string covered terms related to human milk and maternal dietary intake, such as: (“breast feed*” OR breastfeed* OR “breast fed*” OR breastfed* OR lactat* OR “mother* milk” OR “maternal milk” OR “human milk” OR “breast milk” OR breastmilk) and (“maternal diet*” OR “maternal food*” OR “maternal nutrition*” OR “mother* nutrition*” OR “mother* food*” OR “mother* diet*”). Additionally, some publications were included through snowballing.

### Publication screening and inclusion

Articles were screened in duplicate for eligibility through title and abstract screening, which was followed by full-text screening. Inclusion and exclusion criteria were applied according to the procedures described by Bravi et al. (9) Articles were eligible when: (1) including quantitative information on both maternal dietary intake and nutrient composition of breast milk, (2) associations or relations were estimated and quantified by effect estimates such as correlation coefficients, means from subgroups, estimates from regression models or *p*-values from comparison tests, (3) conducted among apparently healthy mothers, who were not suffering from major chronic diseases or undernourishment, of healthy term infants, without preterm birth and wasted or stunted infants. Maternal undernourishment was assessed by evaluating the distribution of energy intake and/or BMI and food insecurity percentages, if available. Articles were excluded if they focused on: (1) fortified food or dietary supplements and probiotics, (2) pollutants, toxic metals or contaminants and its transfer from maternal diet to human milk.

### Data collection and presentation

Data of selected publications and their supplementary files were extracted independent and in duplicate by two reviewers. If necessary, authors were contacted for additional information or clarification.

Extracted data included: author and year, country, study design, sample size, average age, type of milk, moment of milk collection, exposure and type, outcome and type, dietary assessment method, measure of association (correlation, regression coefficient etc.), results, measure of significance and possible adjustments of analysis.

To determine appropriate correlation cut-offs for our review, we adopted standards that are commonly used in the evaluation of dietary assessment methods. Such evaluation studies typically focus on self-report-based dietary intake methods, which often share correlated errors, potentially inflating observed correlations. As suggested by Willett (13) correlations between two self-report dietary assessment methods should typically fall within the range of 0.5–0.7 for validity. To mitigate the impact of correlated errors, he recommends a minimum cut-off of 0.5 to ensure a correlation of at least 0.4, which is considered moderate. Our analysis involves exploring correlations between self-reported data (e.g., FFQ, recall) and a biochemical measure (breastmilk composition). In this specific context, the likelihood of correlated errors influencing the correlations is significantly lower. Moreover, it’s important to note that our primary focus is on investigating correlations between dietary intake and milk composition, rather than method validation, within a context influenced by various physiological processes. In conclusion, this guided our decision to establish a correlation value of |r| > 0.4 as the suitable threshold for defining satisfactory correlations, 0.2 ≤ |r| ≤ 0.4 as moderate correlations, and |r| < 0.2 as weak correlations. As the cut-off values are adapted from the interpretation criteria for validation of dietary intake assessment methods (13), these should be interpreted with caution. Due to the large number of different fatty acids, only data of the major dietary fatty acid categories are shown in this review, i.e., saturated fatty acid (SFA) (lauric acid, myristic acid, palmitic acid, stearic acid and vaccenic acid), monounsaturated fatty acid (MUFA) (oleic acid, palmitoleic acid), total polyunsaturated fatty acid (PUFA), n-3 PUFA [α-linolenic acid (ALA), DHA (docosahexaenoic acid), EPA (eicosapentaenoic acid), docosapentaenoic acid (DPA)], n-6 PUFA [LA (linoleic acid), AA (arachidonic acid)] and trans fatty acids (elaidic acid and rumenic acid).

The results of this updated review have been combined with those results gathered by Bravi et al. All data was tabulated, including the exposure and outcome type, sample size, measure of association, result and significance of results. There was no missing data after data was tabulated. The combined results are visualized by displaying the associations and correlations between maternal intake nutrients and products with breast milk components, including presentation of the study quality and statistical significance (*p* < 0.05).

This review is conducted following PRISMA guidelines.

### Risk of bias assessment

All included studies underwent a risk of bias assessment, based on an adjusted version of the Study Quality Assessment Tools for cross-sectional studies and controlled intervention studies, developed by the National Heart, Lung, and Blood Institute.^1^ Two researchers independently rated the studies on 11 criteria. For observational studies, this included judgment of the study population, exposure assessment, including used assessment tools and outcome assessment, milk sampling procedure as well as study funding. Trials were rated on their blinding and randomization, study population, follow-up, outcome assessment and funding. Generally, the maximum score was 22 points. However, for six studies the validation of tools was not applicable, and for those studies the maximum score was 20. To allow comparison between studies, the percentage of the maximum score per study is calculated (Supplementary Tables S1, S2).

## Results

**Figure 1:**
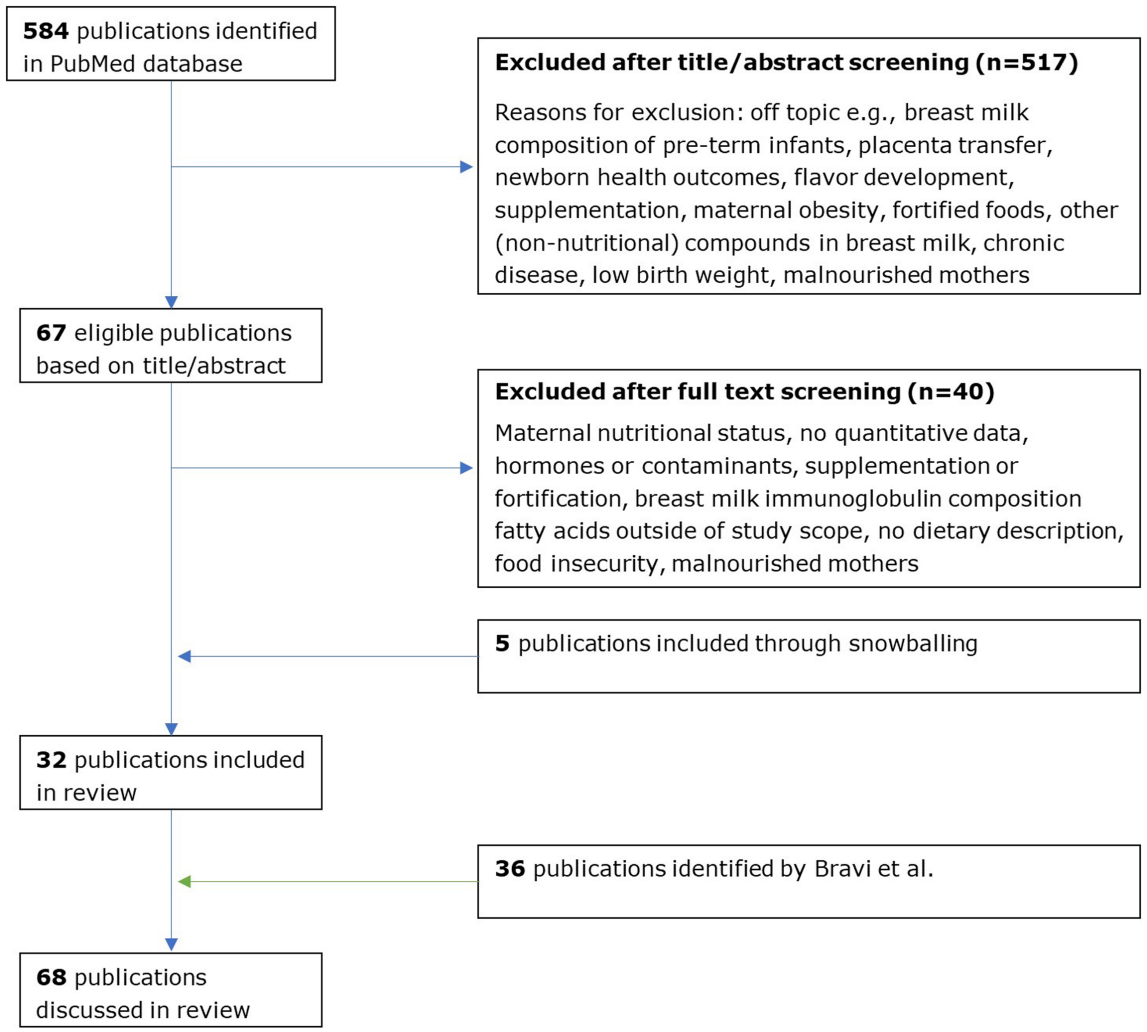
Flow chart of study selection.

A total of 584 articles were identified: 67 articles passed the title/abstract screening, 27 articles passed the full-text screening, and 5 additional articles were identified through snowballing. Eventually, 27 observational studies and 5 intervention studies met the inclusion criteria of this review (Figure 1). To present a complete overview of the available evidence, those papers were combined with the 36 studies identified by Bravi and colleagues. If not stated explicitly, the reported findings result from a study with an observational study design. Details on study design are summarized per study in Supplementary Table S3 for intervention studies and Supplementary Table 4 for observational studies.

**Figure 2:**
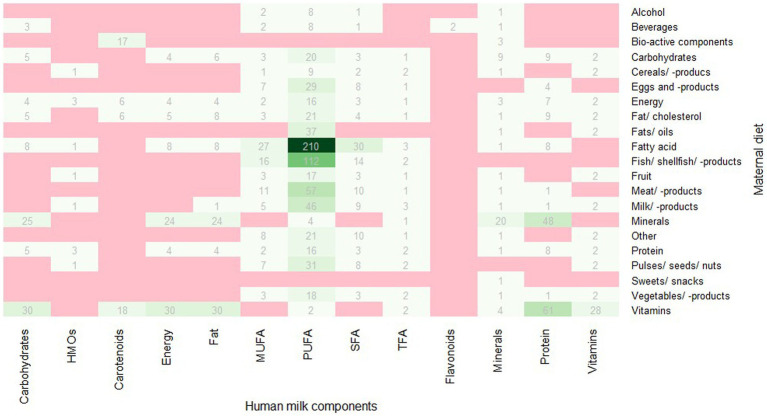
Number of studied associations per outcome and exposure pair, red = no associations studied, darker green = more studied associations. HMOs, human milk oligosaccharides; TFA, trans-fatty acid.

Most articles (*n* = 19) provided information related to more than one of the following nutritional compounds, i.e., 2 articles provided data on human milk energy content, 7 articles on protein content, 5 articles on fat content, 10 articles on SFA content, 11 articles on monounsaturated fatty acid (MUFA) content, 15 articles on polyunsaturated fatty acid (PUFA) content, 5 articles on trans fatty acids (TFA) content, 6 articles on carbohydrate content, 5 articles on vitamin content, 5 articles on carotenoids, 5 articles on mineral content, and 1 article on flavonoid content. In total, 1,490 associations were assessed (Figure 2).

### Energy

Bravi and colleagues identified two studies on maternal dietary intake and human milk energy content; one suggested a significant association between higher milk energy content for a high-fat/low-carbohydrate diet (9).

Our updated search resulted in the identification of two new studies. Significant positive associations were observed for the intake of fat, cholesterol and MUFA (*r* = 0.13; *r* = 0.18, and *r* = 0.15, with milk energy content, respectively), among 238 South Korean women, whereas no associations were observed for dietary intake of energy, carbohydrate, protein, SFAs and PUFAs, and milk energy content (16). No associations between maternal intakes of fat, energy, protein, carbohydrate, minerals and vitamins and milk energy content were reported among Polish women (17).

### Protein

Bravi and colleagues identified four studies exploring the role of maternal dietary intake in human milk protein content, showing one significant association between a higher human milk protein content and a maternal high-protein diet (9).

Our updated search resulted in the identification of seven new studies. No significant correlations were observed between maternal protein intake and milk total protein content (18), nor between the intake of protein, energy, carbohydrate, fat, minerals, vitamins, cholesterol, SFA, MUFA and several PUFAs, and milk protein content (16, 17). A recent trial among 10 Australian mothers did not show an effect of consumption of a high-sugar diet on milk protein content in the 12 h assessed afterwards (see Supplementary Table S4), but the high-fat diet resulted in slightly lower milk protein levels compared to the control group (15). In addition, a New Zealand based study reported moderate inverse correlations between maternal intake of vitamin D and starch intake with milk protein content (both *r* = −0.2 to −0.4), however no level of significance was given (19).

Besides that, among Chinese mothers, no significant associations were observed between intake of meat, dairy, eggs, and vegetables and milk lactoferrin content (20). However, in a randomized controlled trial among 120 Australian mothers, after a 6-week high-egg diet, milk ovalbumin was significantly higher, when compared to a low-egg and egg-free diet (0.20 vs. 0.05 vs. 0.05 ng/mL, *p* = 0.04), whereas no difference were observed after 2 and 4 weeks (21).

### Total fat

Bravi and colleagues identified seven studies on the association of dietary intake and total fat content, three of them showed significantly higher human milk fat concentrations with higher intakes of fat, dairy or phytosterol (9).

Our updated search resulted in the identification of five new studies on this topic with significant associations being observed by both identified intervention studies. A US based cross-over study among 15 women (see Supplementary Table S4) showed that a full-fat dairy diet for 14 days resulted in higher milk fat content compared to the low-fat diet (3.35 vs. 2.41 g/100 g milk, respectively) (22). In the Australian trial of Ward and colleagues (see Supplementary Table S4), a high-fat diet (69.3 g sugar/day, 129.7 g fat/day) and a high-sugar diet (150.4 g sugar/day, 77.5 g fat/day) increased milk triglyceride content, compared to the control diet (83.7 g sugar/day, 101.5 g fat/day) (mean difference of 3.05 and 13.8 g/dL, respectively). Human milk cholesterol was only increased with the high-sugar diet, when compared to control (15). Positive correlations were reported in a South Korean cohort study for maternal fat, MUFA and cholesterol intake with milk fat content (*r* = 0.14; 0.15, and 0.18 respectively) (16). In contrast, no correlations were observed between maternal fat intake and milk fat content in cohort studies performed in Latvia and Poland (17, 18). No associations between maternal intake of energy, carbohydrate, protein (16, 17), mineral, vitamin (17), SFA, PUFA, n-3 PUFA, n-6 PUFA, AA, EPA, and DHA (16) with milk fat content were seen.

### Fatty acid

**Figure 3:**
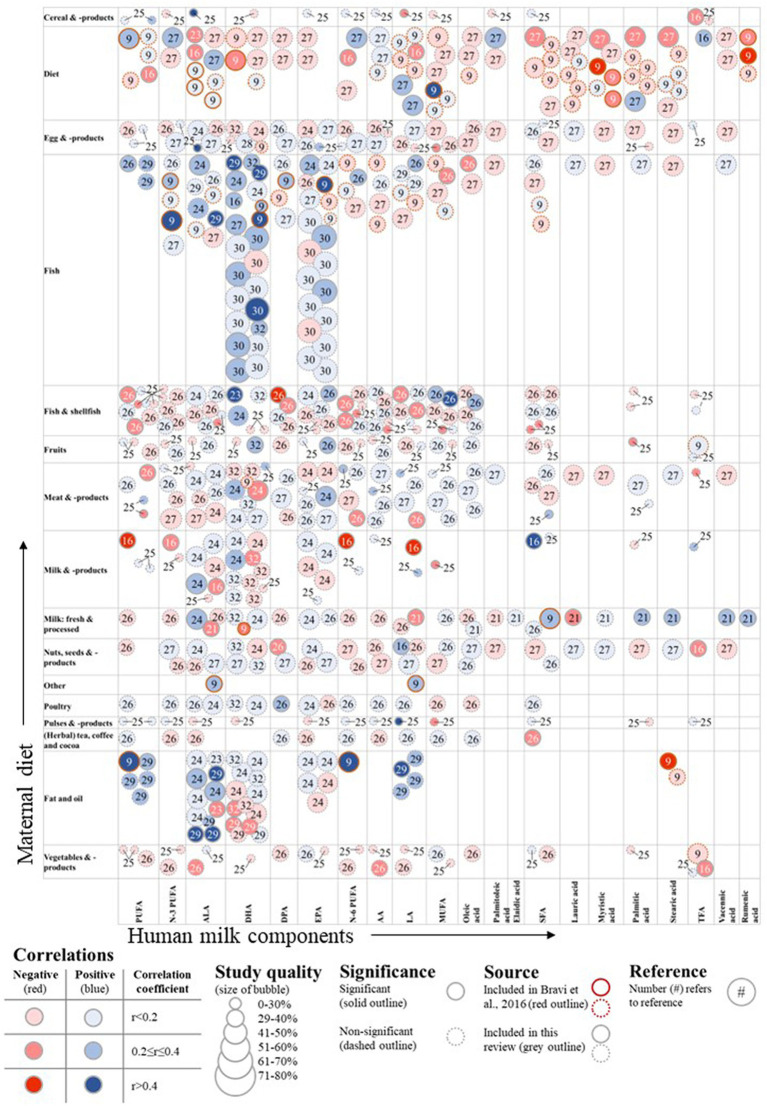
Associations of maternal diet and human milk fatty acid composition. DPA, docosapentaenoic acid; TFA, trans-fatty acid. *The label diet includes dietary patterns that include more than one food group or a calculated index, including (dairy) Healthy Eating Index (HEI) (14), high-fat or high-sugar diets (15).

**Figure 4:**
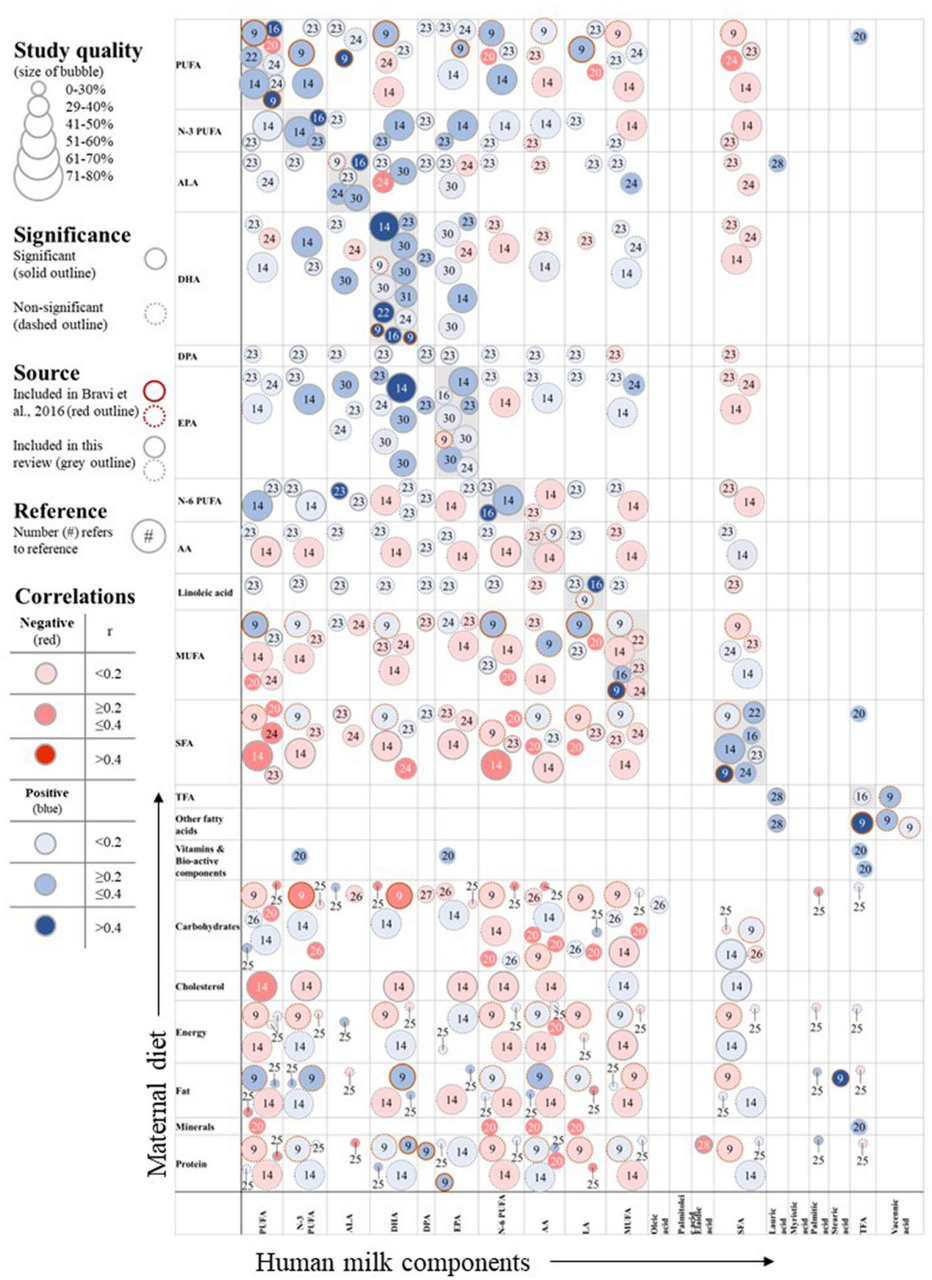
Associations of maternal nutrient intake and human milk fatty acid composition. DPA, docosapentaenoic acid; TFA, trans-fatty acid.

The available evidence on maternal diet and human milk fatty acid concentration is summarized in Figures 3, 4 and also include the results of Bravi et al. The figures show the identified associations per exposure and outcome combination, including the study quality as well as direction and strength of association. Note that various studies described multiple associations and are depicted by multiple circles referring to different results from the same study.

### Saturated fatty acid

Six publications on the association of diet and SFA content were previously identified by Bravi et al., of which two showed significant correlations between SFA and high-fat dairy intake and human milk SFA content (9).

Since 2015, eight new studies explored this topic, in which 14 statistically significant associations were reported (Figures 1, 2). Positive correlations between SFA intake and milk SFA concentrations were observed among Chinese, South Korean and French women (16, 23, 24). No associations between maternal MUFA, PUFA, ALA, EPA, and DHA intake and milk SFA content were observed by the Polish and South Korean cohorts (16, 25). In the French EDEN cohort, Armand and colleagues observed significant negative correlations between PUFA, n-6 PUFA, LA, and n-3 PUFA intake and SFA content (24), while Kim et al. observed no significant correlations between the intake of MUFA and several PUFAs and milk SFA content among 238 South Korean women (16). Similarly, no associations between maternal intake of several fatty acids and milk SFA were observed in the cohort of 32 Polish women (Figure 2) (25).

In the South Korean study of Kim et al., energy, carbohydrate and cholesterol intake were positively associated with milk SFA content, while protein and fat intake was not (16). Similarly, among 65 Chinese women, no significant associations for the intake of macronutrients with milk SFA content were seen (Figure 2) (26).

When examining food groups, milk and dairy intake showed satisfactory positive correlations with milk SFA content among 61 Latvian women (18), while a moderately significant positive correlation between intake of meat and milk SFA content was observed among a Chinese cohort (26). Besides that, only coffee and tea intake was negatively associated with milk SFA concentrations (27). All other assessed food groups did not show any significant correlations with milk SFA content (Figure 1) (14, 18, 26, 27).

In terms of dietary patterns, the Canadian CHILD cohort study of 1,094 women observed an inverse correlation between the Healthy Eating Index (HEI) diet quality score and milk SFA content (*β* = −0.01 to −0.50) (14).

### Lauric acid (C12:0)

Of the four previously identified studies, one study indicated a significant association between a low-fat diet and human milk lauric acid content (9).

During the past 5 years, three new studies explored this topic and reported four significant associations (Figures 1, 2). The Polish observational study by Bobiński et al. showed positive correlations between lauric acid, TFA and ALA intake and milk lauric acid content (28).

In terms of food groups, no associations were observed in the Canadian CHILD cohort (14). Yet, in a US based randomized cross-over study (see Supplementary Table S4), a low-fat dairy intervention resulted in higher milk lauric acid content compared to a high-fat dairy dietary intervention after 14 days (5.26 vs. 4.58 g/100 g fat respectively) (22). Considering diet quality, HEI was not associated with milk lauric acid content (14).

### Myristic acid (C14:0)

Three out of six previously identified studies observed a significant association between hydrogenated fat, an organic diet or high-dairy diet and higher human milk myristic acid concentrations (9).

Since 2015, two new studies explored this topic (Figure 1), in which one statistically significant association was reported. In terms of diet quality, the CHILD cohort observed an inverse association between HEI and milk myristic acid content (*β* = −0.01 to −0.50), but not for the consumption of certain food groups or the dairy specific HEI (14). Similarly, the cross-over study among 15 women living in the US (see Supplementary Table S4) reported that the consumption of a high- and low-fat dairy diet did not result in a difference in milk myristic acid content (22).

### Palmitic acid (C16:0)

Six studies on human milk palmitic acid content were identified by Bravi et al. Two of them observed a significant positive association between a high-dairy diet or a nonhydrogenated fat diet and human milk palmitic acid content. Another study reported that a meal with a high PUFA-to-SFA ratio resulted in lower palmitic acid concentrations (9).

In the three newly identified studies, 6 significant associations were reported (Figures 1, 2). Positive and inverse correlations were observed between protein and carbohydrate intakes and milk palmitic acid content among 65 Chinese mothers, respectively (26).

In terms of food groups, a correlation between intake of fruit and milk palmitic acid, was reported, while other food groups showed no significant correlations (26). In line with the latter, no significant associations were observed for several food groups as part of the CHILD cohort (14).

Considering dietary patterns, significant associations were reported for the association with milk palmitic acid content. An inverse correlation was observed between HEI (*β* = −0.01 to −0.50) and a positive correlation for dairy HEI, reflecting only an effect of the diet quality of dairy products component (*β* = 0.01–0.5) in the CHILD cohort (14). In addition, in the cross-over study of Yahvah et al. (see Supplementary Table S4), a high-fat dairy diet resulted in higher milk palmitic acid content compared to a low-fat dairy diet fed for 14 days (21.62 vs. 19.29 g/100 g fat, respectively) (22).

### Stearic acid (C18:0)

Two out of six previously identified studies showed a significantly positive association between a high-fat diet or a high-dairy diet and human milk stearic acid content and a high PUFA/SFA ratio meal resulted in lower proportions of stearic acid (9).

Only one of two newly identified studies focused on the association of intake of several food groups and human milk stearic acid, but no significant associations were observed (Figure 1) (14).

Two studies were performed on diet quality and reported two statistically significant associations. An inverse association between HEI and milk stearic acid content (*β* = −0.01 to −0.50) was observed in the CHILD cohort study, while no significant association was observed for the dairy HEI (14). However, a causal relation with dairy was seen in a 14 days cross-over trial. A high-fat dairy intervention (see Supplementary Table S4) resulted in a higher milk stearic acid content compared to a low-fat dairy diet (7.5 vs. 5.8 g/100 g fat, respectively) (22).

### Monounsaturated fatty acid

Bravi and colleagues identified six studies that showed a significant positive correlation between MUFA intake and human milk content as well as a negative correlation between an organic diet and MUFA concentrations (9).

The updated search identified nine new studies, reporting 9 statistically significant associations (Figures 1, 2). A positive association between MUFA intake and milk MUFA content was observed among 61 Latvian women (18). In contrast, other studies did not see any association between maternal MUFA intake and milk MUFA content (16, 23–25). The same group of authors studied intake of several other fatty acids, but none of them was significantly associated with milk MUFA concentration (Figure 2) (16, 24, 25).

Inverse correlations between energy and carbohydrate intake and milk MUFA content were observed, but no significant correlations were observed for protein, fat and cholesterol intake (16). Similarly, in the Chinese study of Deng et al., no significant associations for maternal nutrient intake and milk MUFA content were seen (26). Among 78 women in New Zealand moderate inverse correlations for maternal carbohydrate intake and milk MUFA were reported (*r* = −0.2 to −0.4), yet, the level of significance is unknown (19).

In terms of food groups, Jagodic observed a significant positive association with maternal intake of (fresh) seafood (27). However, in the same study, as well as in the study of Deng and colleagues significant inverse associations for other seafood groups were seen, namely freshwater fish (26) and fish and shrimp (25). Additionally, Deng and colleagues observed a significant inverse association with the maternal intake of eggs and dairy products (26). Other studies did not observe associations between intake of certain food groups and milk MUFA concentration (Figure 1) (14, 26, 27).

### Palmitoleic acid (C16:1n7)

All four previously identified intervention studies indicated a significant positive relation between a low-fat diet as well as a nonhydrogenated fat diet and human milk palmitoleic acid content (9).

While no significant correlations were observed for several nutrients and food groups by three newly identified studies (Figures 1, 2), a positive correlation was observed between dairy HEI score and milk palmitoleic acid content in the CHILD cohort (14). However, a high-fat dairy and low-fat dairy dietary cross-over intervention (see Supplementary Table S4) did not result in a difference in milk palmitoleic content (22).

### Oleic acid (C18:1n9)

Two out of five studies included in Bravi’s review showed a significant positive association between a hydrogenated fat or high-dairy diet and oleic acid content. A diet with a high PUFA-SFA ratio resulted in a lower oleic acid concentration (9).

Since then, three new studies explored this topic, with two significant associations reported (Figures 1, 2). Miliku et al. did not observe any significant association of maternal food or nutrient intake and milk oleic acid content (14). In a Slovenian study among 74 women, no associations were observed between intake of several food products with milk oleic acid concentration (Figure 1). However, a negative association of fresh water fish intake, and a positive association of fresh seafood intake with milk oleic acid content were reported (27).

In contrast to the results in Bravi’s review, a high-fat- and a low-fat-dairy dietary cross-over intervention (see Supplementary Table S4) did not result in a difference in milk oleic acid (22).

### Polyunsaturated fatty acid

Four out of five studies included in the Bravi et al. review indicated significant positive associations between MUFA, PUFA, diet quality index as well as vegetable oil-based spreads and human milk PUFA concentration (9).

Nine new studies were identified in the updated search and reported a total of 26 significant associations (Figures 1, 2). Four studies observed significant positive correlations between PUFA intake and human milk PUFA content (16, 18, 23, 24). Additionally, a South Korean and a French study reported positive associations between n-6 PUFA, n-3 PUFA intake and milk PUFA concentration (16, 24). In contrast, Bzikowska-Jura et al., did not observe a significant correlation in their study among 32 Polish women (25).

Inverse correlations were reported between the intake of SFA and AA with milk PUFA (16). Similarly, SFA intake was negatively correlated with milk PUFA in the French EDEN cohort (24). In the same study, LA, ALA, and MUFA intake were positively associated with PUFA content, while no significant associations were observed for AA, EPA, DPA, and DHA intake and milk PUFA content (24). The New Zealand cohort of Butts et al. saw inverse associations of maternal PUFA, MUFA, saturated fat, carbohydrate, and sodium intake with milk PUFA content, yet the level of significance is unknown (19). Kim et al. observed an inverse correlation between cholesterol intake and milk PUFA content, whereas no significant associations were observed for energy, carbohydrate, protein, fat, EPA and DHA intake (16). Furthermore, the Chinese study of Deng and colleagues observed a positive association for the intake of carbohydrates and a negative association for the intake of fat and protein with milk PUFA content (26).

In terms of food groups, negative associations with milk PUFA content were reported for milk and dairy consumption, and eggs and meat in a cohort of Latvian women (18). In line with that, meat intake was negatively correlated with human milk PUFA content in the study of Deng et al., but no significant associations were observed for several other foods, including fish & shrimp and seafood intake (Figure 1) (26). Contrarily, a study among Slovenian women identified a positive association between maternal freshwater fish intake, but a negative association between maternal (fresh) seafood intake with milk PUFA content. Game intake was negatively associated with milk PUFA, but there was not an association shown for the intake of poultry, other meat, nor for vegetable, fruit, milk, nut, egg and frozen/canned seafood intake (27). In the Swedish FARMFLORA birth cohort, Jonsson et al. saw a significant positive correlation between margarine and oil intake and milk PUFA content, whereas no significant associations were observed for margarine and fatty fish intake during pregnancy and fatty fish, margarine and oil and margarine intake during the fourth month of lactation (29).

### N-3 polyunsaturated fatty acid

Bravi et al. reported three studies, indicating a significant association between intakes of PUFA as well as fatty fish and human milk n-3 PUFA content (9).

Since then, seven new studies explored this topic and reported 18 significant associations (Figures 1, 2). The intake of n-3 PUFA was positively associated with milk n-3 PUFA concentrations in three studies (16, 18, 24). In addition, among South Korean and French women positive correlations between intakes of PUFA, n-6 PUFA, EPA, DHA, LA, ALA, and DPA and milk n-3 PUFA concentration were observed (16, 24). Inverse correlations were reported between SFA intake and milk n-3 PUFA content, whereas no correlations were observed for energy, cholesterol, carbohydrate, protein, fat, MUFA and n-3 FA intakes and milk n-3 PUFA content (Figure 2) (16).

Among 78 New Zealanders, maternal intake of vitamin A equivalents was positively moderately associated with milk n-3 PUFA, however no significance was provided (19). Conversely, no significant correlations were observed between various macronutrients and food groups and milk n-3 PUFA content among Chinese women (26).

Considering food groups, one study observed a negative association of maternal milk and dairy intake and human milk n-3 PUFA content (18). No other food groups were associated with milk n-3 PUFA content (Figure 1) (14, 27), while the CHILD cohort reported a positive correlation between HEI and human milk n-3 PUFA (*β* = 0.01–0.50) (14).

### α-linolenic acid (C18:3n3)

Four out of eight previously identified studies showed significant positive associations between PUFA and PUFA-to-SFA ratio, a non-organic diet, a low-dairy diet as well as a high-fat diet and human milk ALA content (9).

Since 2015, nine new studies explored this topic, reporting a total of 28 significant associations (Figures 1, 2). ALA intake was significantly positively associated with milk ALA content in three studies (18, 24, 30). In addition, intake of PUFA, n-6 PUFA, LA (24) as well as EPA and DHA (30) were positively correlated with milk ALA content, while SFA intake was negatively correlated (24). No associations were observed for MUFA, AA, n-3 PUFA, EPA, DHA, and DPA intake in a French cohort (24).

In two studies, energy intake was positively correlated with milk ALA content, but no nutrients or bioactive compounds showed any association (Figure 2) (26, 27).

In terms of food groups, satisfactory and moderate positive correlations were reported for the intake of linseed oil and coconut oil (25), and margarine and oils (29) and human milk ALA concentration. In contrast to that, coconut butter intake was negatively associated with milk ALA in the study of Armand et al. (24). Milk, as well as milk products showed mixed results, with both, moderate positive (25) as well as moderate negative associations in the cohort of Aumeistere (18). In the trial of Yahvah and colleagues (see Supplementary Table S4), a full fat dairy interventional lead to lower ALA concentration, compared to a low fat diary diet (22). Fish (25, 29), egg and cereal intake (26) showed moderate to satisfactory positive associations with milk ALA content. No significant correlations were observed for any other food group (Figure 1).

In the French EDEN cohort, cheese, added fat, fresh and dried fruit intake were associated with human milk ALA content (*β* = −0.001) (24). Aumeistere et al. saw associations for egg and meat intake with milk ALA content (*r* = −0.32) (18). The Canadian CHILD cohort study reported a positive correlation for HEI during pregnancy (*β* = 0.01–0.50) and milk ALA (14).

### Docosahexaenoic acid (C22:6n3)

In the report of Bravi, four out of eight included studies observed significant positive associations between intakes of protein, fat, PUFA, fish and fatty fish and milk DHA content. One study reported inverse associations between carbohydrate intake and human milk DHA content (9).

In the updated search, 11 new studies were identified, with 36 statistically significant associations reported (Figures 1, 2). Human milk DHA concentration was significantly positively correlated with maternal DHA intake in six studies (16, 18, 23, 24, 30, 31). Only few studies observed an insignificant association (25, 30). Furthermore, positive correlations were observed for maternal ALA (24, 30), DPA (24), EPA (16, 24) and n-3 PUFA (16, 24) intake and DHA concentration in milk. Significant negative weak associations were reported for maternal MUFA and SFA intake and the concentration of DHA in milk (16, 24, 30). Maternal cholesterol intake was also negatively correlated with DHA content in milk (Figure 2) (16).

Considering food groups, six studies observed moderate positive correlations with maternal fish intake (14, 18, 25, 30, 32). In addition, among 803 French women a satisfactory association of maternal fish and shellfish consumption and milk DHA was reported (24). Besides fish, avocado intake was moderately positively associated with milk DHA level (32), while margarine (29) and beef (25) were negatively correlated with milk DHA. Other food groups did not show any significant correlations (Figure 1) (14, 25, 26, 29, 30, 32).

### Eicosapentaenoic acid (C20:5n3)

Regarding human milk EPA content, two out of four previously included studies observed significantly positive associations between PUFA, protein as well as fatty fish intake and human milk EPA content (9).

In the updated search nine new studies were identified with 16 significant associations reported (Figures 1, 2). Maternal EPA intake was moderately positively correlated with its milk content in two studies (16, 24), all other studies presented non-significant results (Figure 2) (18, 25, 30). Armand et al. and Kim et al., also reported significantly positive correlations between maternal ALA, DHA, DPA, and n-3 PUFA intake with milk EPA content. Intake of SFA and cholesterol was associated with lower milk EPA level (16, 24).

The effect of food groups on milk EPA content appeared limited. While the association of fish and the combined group of fish and shellfish were studied frequently, only four moderately positive associations were reported (25, 27, 30). Besides, fish, fruit and pork consumption showed to be moderately positively associated with milk EPA (25, 27). All other studied associations showed no significant results (Figure 1) (14, 25–27, 30). Riboflavin intake was moderately positively correlated with EPA in milk, but no level of significance was given (19).

### Docosapentaenoic acid (C22:5n3)

Bravi et al. identified two studies on the topic of human milk DPA content, both observed significant positive associations between protein intake or fatty fish intake and human milk DPA content (9).

Three studies were added in the updated search, reporting 10 statistically significant associations (Figures 1, 2). Moderate positive correlation were reported for maternal EPA and DHA intake, while ALA, DPA and n-3 PUFA intake were weakly positively correlated with DPA in milk. MUFA intake was negatively correlated with milk DPA content (24).

Among Slovenian mothers, consumption of fresh seafood and nuts were negatively correlated with milk DPA content, while a positive association was reported for poultry intake (27). Yet, in the Canadian CHILD cohort, fish and several other foods did not show any association (14).

### N-6 polyunsaturated fatty acid

Two out of three studies identified by Bravi and colleagues on n-6 PUFA content indicated significant positive associations between MUFA and vegetable oil intake and human milk n-6 PUFA concentration (9).

Since 2015, seven new studies on that topic were published, which reported 19 significant associations (Figures 1, 2). Maternal intake of n-6 PUFA was positively correlated with its content in milk in all three studies on that association, with varying strength of association (Figure 2) (16, 18, 24). Besides that, n-6 PUFA in milk was shown to be positively associated with maternal intake of LA, MUFA, and PUFA (16, 24). Negative correlations were reported for the intake of AA and SFA (16). Among women in New Zealand, moderately negative associations between the intake of MUFA, PUFA, and SFA with milk n-6 PUFA were observed, however no level of significance was given (19).

Maternal cholesterol intake was negatively correlated with n-6 PUFA concentration in milk among South Korean women (16), while no other correlations with nutrients were seen.

Looking at food groups, maternal fish intake was reported to be moderately positively associated with milk n-6 PUFA content (27). Interestingly, the combined group of fish and shellfish intake showed moderate negative correlations with milk n-6 PUFA content (27). Results for maternal meat intake are contradicting, with a Chinese study reporting a moderate positive association (26) and a Slovenian study a moderate negative association (27). Milk and dairy intake showed satisfactory negative correlations with human milk n-6 PUFA (18). No other food group showed significant associations with n-6 PUFA content in milk (26, 27).

The combined variable of meat and egg was inversely associated with n-6 PUFA in milk among Latvian women (18). In the Canadian CHILD cohort, one positive correlation was observed between HEI and milk n-6 PUFA content (*β* = 0.01–0.50) (14).

### Linoleic acid (C18:2n6)

Only four out of 11 previously identified studies showed significant positive associations between intake of a high PUFA-to-SFA ratio, MUFA, PUFA, olive oil and human milk LA content (9).

The updated search identified eight new studies, reporting a total of 21 significant associations (Figures 1, 2). In the newly identified studies, the intake of LA was positively associated with its level in milk in two studies (18, 24). Further, the intake of MUFA, n-6 PUFA and PUFA were positively correlated with milk LA content, while SFA intake was negatively associated (24). Among Chinese women carbohydrate intake was found to be positively and fat intake negatively correlated with the level of LA in milk (26). And while Butts et al. reported inverse associations of maternal PUFA, MUFA, saturated fat, carbohydrate, and sodium intake with milk PUFA content, the level of significance is unknown (Figure 2) (19).

A Slovenian study reported positive and inverse correlations with consumption of game, fresh water fish and fresh seafood and milk LA content (27). In addition, nuts and seeds and pulses (18, 26) as well as margarine and oils (29) showed to be positively associated with LA in milk. In contrast to that, in the study of Miliku et al., nut intake showed no significant association (14). Human milk LA levels showed moderate to satisfactory negative correlations with intake of milk and milk products (18) and cereals (26).

Considering diets, the combined variable of meat and egg was inversely associated with LA in milk in a Latvian study (18). Among Canadian women, total and dairy HEI was positively associated with LA in milk (14). Yahvah et al. observed in their cross-over study that a high-fat dairy diet intervention resulted in lower human milk LA content compared to a diet with low-fat dairy diet fed for 14 days (14.27 vs. 18.80 g/100 g fat, respectively) (see Supplementary Table S4) (22).

### Arachidonic acid (C20:4n6)

Of the five previously reported studies, one observed a significantly positive association between a low-fat diet and human milk AA concentration (9).

Since 2015, six new studies explored this topic, with one significant association being reported (Figures 1, 2). Maternal intake of SFA was shown to be negatively associated with milk AA content (16). In addition, among women in New Zealand negative correlations between intake of protein, sugars, energy, carbohydrate and sodium intake with milk AA concentration were reported, yet the level of significance is unknown (19). Other studies however did not observe any significant correlations (14, 24, 26, 27).

### Trans fatty acid

During the previous search, only one study was identified on TFA content, which showed a significantly positive association with intake of elaidic acid (9).

The updated search resulted in three new studies, reporting 4 statistically significant associations (Figures 1, 2). Butts et al., reported positive correlations of maternal PUFA, calcium, riboflavin, retinol and saturated fat intake and milk TFA (19). Aumeistere and colleagues did not see a significant correlation between TFA dietary intake and milk TFA content, yet a positive association for egg and meat intake and inverse association for grains and cereals, vegetables and legumes and nuts and seed intake were observed with milk TFA (18). On the contrary, no significant correlations were observed by Deng et al. between energy, the macronutrients, cereal, vegetables, fruit, meat, dairy, bean, egg and seafood intake and milk TFA content (Figure 1) (26).

### Elaidic acid (C18:1n-9t)

Before our search, one intervention study was identified about elaidic acid content, showing that a hydrogenated fat diet resulted in higher human milk elaidic acid content (9).

Our updated search identified two studies focusing on animal-based product consumption and milk elaidic acid content (Figures 1, 2). An inverse correlation between intake of dietary animal protein during pregnancy and milk elaidic acid content was observed by Bobiński et al. (28), while no difference between low-fat dairy and high-fat dairy diet in a 14-day cross-over was shown (*p* > 0.05) (see Supplementary Table S4) (22).

### Rumenic acid (C18:2n7t,9c)

Both studies identified by Bravi and colleagues indicated positive associations between an organic diet, a high-dairy diet, rumenic acid and MUFA intake and rumenic acid concentrations in human milk (9).

Since 2015, only one cross-over study explored the topic (Figure 1). Yahvah et al. observed that a high-fat dairy diet intervention resulted in higher human milk rumenic acid compared to a diet with low-fat dairy (0.33 vs. 0.24 g/100 g fat respectively) (see Supplementary Table S4) (22).

### Vaccenic acid (C18:1n7t)

One out of two previously identified studies indicated a significant association between an organic diet and human milk vaccenic acid content (9).

Since then, two additional studies explored this topic, with one significant association reported (Figure 1). The association of several food groups was studied among Canadian women, but no significant association was found (14). Yahvah and colleagues observed that a high-fat dairy intervention resulted in higher milk vaccenic acid content compared to a low-fat dairy diet (0.54 vs. 0.34 g/100 g fat, respectively) (22).

### Total and individual carbohdrates

Bravi et al. identified four studies, none showing significant associations (9).

Our updated search resulted in the identification of six new studies on this topic. Butts et al. indicated associations between maternal zinc and protein intake and milk carbohydrate content (*r* = 0.2–0.4, no level of significance given) (19). Bzikowska-Jura et al. could not confirm these results and observed no significant correlations for intakes of energy, the macronutrients, and several minerals and vitamins with milk carbohydrate content, irrespective of the time post-partum (1st, 3rd, and 6th month sampled) (17).

In a cross-over study among 41 American women, intake of a high-fructose corn-syrup-sweetened (HFCS) beverage resulted in significantly higher milk fructose concentrations compared to the no caloric sugar/artificially sweetened control beverage up to 5 h after ingestion (33). No correlation was observed among Latvian women between maternal lactose intake and milk lactose content (18) and Kim et al., who studied the association of energy, macronutrients, MUFA, PUFA, SFA, and cholesterol intake with milk lactose concentration did not observe any associations either (16).

Azad and colleagues studied associations of maternal diet and human milk oligosaccharides (HMOs) in milk in the Canadian CHILD cohort. After adjusting for multiple comparisons, no associations were observed between seafood and plant protein, total calories, fatty acids, greens and beans, whole grains and fruit and human milk levels of several individual HMO and HMO diversity (34).

### Vitamins

Bravi and colleagues identified six studies, of which two studies indicated a significant positive association between vitamin C intake and one study between vitamin E, thiamin and riboflavin and its respective milk levels. Another study reported significant inverse associations between fat and SFA intake and milk vitamin E content (9).

Our updated search resulted in the identification of five new studies on this topic. Among 113 Indonesian women, positive associations were observed between maternal vitamin A, retinol, niacin, and riboflavin intakes and their respective levels in milk, which increased by 23.5% (95% CI: 1.2–50.7), 28.8% (95% CI 0.45–52.5), 4.0% (95% CI 0.49–7.6), and 79.1% (95% CI 21.2–165.0), respectively per unit increase in their intake. However, no significant associations were observed for maternal intakes of thiamin, vitamin B6 and vitamin B12 with their concentration in milk (35). Vitamin A intake was positively associated with milk retinol in a study of 19 Brazilian women, both, after 2–4 weeks postpartum (*β* = 0.005, *p* < 0.001) and after 12–14 weeks (*β* = 0.005, *p* < 0.001) (36). An observational study in Indonesia additionally reported a positive association of vitamin B3 (niacin) intake and milk nicotinamide concentration in the second month postpartum only, with a mean difference of 3.9% for each unit greater intake (95% CI 1.6–6.2), no further associations between vitamin A, vitamin B1 (free and total), vitamin B2 (free and total), vitamin B3 and vitamin B6 with their respected concentration in milk or with milk retinol, were observed (37). No associations were observed for vegan or vegetarian diets and milk choline (38). Another study did not observe significant correlations between grain, vegetables, fruits, bean products, dairy, egg and meat, oil, diet quality, protein, fat, carbohydrate and energy intake on milk retinol and α-tocopherol content among 102 Chinese women (39). A Kenyan study saw no association between vitamin B12 intake and milk vitamin B12 concentration (40).

### Minerals

Bravi and colleagues identified seven studies, three of them indicating significant associations between maternal zinc, rice, as well as energy intake and human milk zinc content. Maternal egg intake or a vegetarian diet was positively correlated with milk selenium content. Energy intake was positively associated with milk iron content (9).

Since 2015, five new studies were published on the topic. Butts et al. reported a positive correlation of thiamin intake and milk calcium (*r* = 0.2–0.4) with unknown significance (19). Human milk calcium was studied by three authors, Gibson et al. observed a weak positive association of maternal calcium intake and milk calcium content with a mean difference of 0.01% per unit increase (95% CI 0–0.03) at 2 months but no significant association at 5 months postpartum (37). Daniels and colleagues did not find a significant association at 2–5.5 months postpartum (35). Maternal iron intake and milk iron content was only associated for the 2nd month postpartum (mean difference 2.4, 95% CI 0.3–4.5), and not after 5 months (35, 37). An association between maternal intake of potassium, folate, magnesium, fiber, iron, caffeine, iodine, starch, sugar, energy and carbohydrate with iron milk levels was reported (all *r* = 0.2–0.4) but no level of significance was given. PUFA and caffeine intake were associated with milk selenium (all *r* = −0.2 to −0.4), but again, the level of significance of those results are unknown (19).

Among Latvian women, no significant correlations between maternal dietary energy, macronutrient, zinc, grains, meat, milk, vegetables/legumes, fruit/berries, oils/shortening, sweets/snacks and caffeine containing drinks consumption and milk zinc content were observed (41). In line with that, Daniels et al. did not observe significant associations between zinc intake and milk zinc content among 113 Indonesians (35), while in the study of Gibson et al., an inverse association was reported for the 2nd month postpartum only (mean difference − 3.9% per unit increase in zinc consumption, 95% CI −6.7 to −1.0) (37). Butts and colleagues reported correlations of maternal intake of vitamin A equivalents, beta-carotene equivalents, magnesium, alcohol, vitamin E, zinc, sugars, energy, and carbohydrate with milk zinc content (all *r* = 0.2–0.4), however no indication of significance was made (19). No associations were observed for maternal potassium intake and milk potassium content (35, 37).

### Carotenoids and flavonoids

Bravi et al. identified no articles on the topic of human milk carotenoids, β-carotene, lycopene, lutein, zeaxanthin or flavonoids content (9).

Since then, six studies focusing on human milk carotenoids content and one study examining flavonoids have been published. Among Polish women, positive associations were observed between carotenoids intake and its corresponding milk carotenoids content for β-carotene, lycopene and combined lutein and zeaxanthin at the third month (*β* = 0.41, *p* = 0.012, *β* = 0.42, *p* = 0.010 and *β* = 0.73, *p* = 0.000, respectively) and at the sixth month (*β* = 0.43, *p* = 0.001, *β* = 0.40, *p* = 0.013, *β* = 0.64, *p* = 0.000, respectively), while no significant correlations were observed for energy, vitamin E and vitamin A intake and milk carotenoids at both timepoints (42). Two other studies confirmed the positive moderate association between maternal lutein intake (*β* = 0.363, *p* = 0.0056) (43) and milk lutein content (*r* = 0.44–0.53, *p* < 0.05) (44).

Machado et al. and Gibson and colleagues reported weak associations of beta-carotene and vitamin A intake and milk beta-carotene (*β* = 0.00003, *p* = 0.04, and mean difference 0.08 with each unit increase in vitamin A consumption, 95% CI 0.02–0.13, at 5 month postpartum) (36, 37). No further associations were observed for vitamin A intake and milk beta-carotene at 2 months postpartum, neither for alpha-carotene and beta-cryptoxanthin (37).

A pilot randomized controlled intervention studied milk flavonoid content. Among 44 German women, a 6-day black tea (2 cups/day) intervention resulted in no detectable genistein and daidzein concentration in milk. However, in the 6-day soy milk intervention (1 serving/day) differences were observed in genistein concentration between baseline (day 1; below detection limit), at day 4 (4.9 nmol/L), 7 (4.3 nmol/L), and 8 (3.5 nmol/L). For daidzein concentration also a decrease was seen from baseline (day 1; below detection limit), to day 4 (9.3 nmol/L), which decreased at day 7 (9.1 nmol/L) and 8 (7.3 nmol/L) (45).

## Discussion

In total, we identified 32 new studies on maternal diet and human milk composition through our updated search, which we combined with the 36 studies identified by Bravi et al. (9). Although several different human milk components were analyzed, studies in healthy lactating women were predominantly focused on human milk fatty acid content. In accordance with the review by Bravi and colleagues, new studies on human milk content of energy, protein, carbohydrates and oligosaccharides, vitamins and minerals were still relatively scarce. Moreover, exposure variables studied were still diverse, including dietary patterns, nutrient intakes, and food group intakes. Thus, although the number of studies examining associations between maternal dietary intake and human milk composition almost doubled since the review by Bravi and colleagues, the number of studies assessing the same exposure and outcome combination was limited.

The association of maternal diet and milk fatty acid profile has been studied most, by far (72% of all studies). Maternal fish intake, the main source of DHA in the human diet, was most convincingly positively associated with milk DHA content. Fat and oil intake primarily showed negative associations with DHA, whereas intake of fat and oil was mainly positively associated with PUFAs in general and omega-3 PUFAs ALA and LA in human milk. On a nutrient intake level, total SFA, total PUFA and all included individual PUFAs from maternal diet were generally positively correlated with their content in milk, except for AA. Consumption of SFA was negatively associated with the concentration of several unsaturated fatty acids in milk, including PUFA, n-6 PUFA, and LA, which may be explained by indirect associations with overall diet quality. Results on all other nutrients in human milk remain inconclusive.

While it is clear that milk lipids originate from diet, body storage and *de novo* synthesis, the main contributing pathway likely differs for specific lipids. To illustrate, only up to 10% of the n-6 PUFA precursor ALA seems to be converted into DHA, which might suggest that dietary DHA is the main source of DHA in milk (46). However, in case of LA and AA, RCT data (*n* = 10) showed that only about 33% of LA and 12% of AA in human milk could be directly linked to the dietary labeled [^13^C]-LA and [^13^C]-AA consumed through a low-fat-diet relatively high in the n-6 PUFA precursor LA (47). Accordingly, previous data also showed that LA and AA are largely stored in adipose tissue before release in the circulation and secretion into milk (47). Moreover, a potential influence of the maternal diet on the human milk long chain PUFAs composition also seems most plausible considering that fatty acids show the largest variation in milk composition as compared to carbohydrates and proteins (48). Specifically, although carbohydrate levels are known to change over the course of lactation, with lower lactose levels in colostrum and increasing levels over time (48), the most obvious variation in milk carbohydrate composition is explained by the mother’s Lewis blood group and secretor status, determining the prominence of 2’-FL, 3-FL and the oligosaccharide profile (49). However, the role of maternal diet in the remaining variation of carbohydrates remains inconclusive and warrants further study. In terms of human milk protein composition, the variation is limited, which suggests that maternal protein synthesis is quite strictly regulated (50). Even under low maternal protein intake, or under very different dietary compositions, milk protein synthesis seems to be retained (this work). This hypothesis is further supported by data showing that compared to the other milk macronutrients, proteins are the least impact by maternal factors in general, also outside the scope of maternal diet (51). However, more variation may be observed when studying protein fractions or amino acids.

Various limitations and strengths related the overall body evidence as well as specifically related to this review warrant some discussion. First, the review by Bravi et al. and consequently this updated review focused on healthy nourished mothers, whereas quite some evidence on the relationship between maternal dietary intake and human milk composition has been gathered from malnourished populations or supplementation studies (10). Yet, to proof if the results of supplementation studies also hold for the general population, larger cohorts with more variation in dietary intake might be needed. When aiming for developing dietary guidelines for lactating women worldwide, it is crucial to, next to maternal dietary intake, consider the (long term) maternal nutrient status, the potential effect and safety of supplementation and the effect on infant growth and development.

Second, current evidence was limited by the diversity in applied methodology. Eighty-six percent of the identified studies were observational in nature, which does not allow causal inference. Moreover, most of these observational studies were cross-sectional studies (14, 16, 18–20, 23–32, 34, 35, 38, 40, 41, 43), where dietary intake and human milk composition were measured at only one time point in a study. However, when it comes to lactation stage, together the studied articles range from the first day up to more than 3 years postpartum (Supplementary Table S3). Additionally, studies substantially varied in sample size, ranging from 19 to 2007 participants. In total, 16 out of 27 observational studies had less than 100 participants, and most studies did not provide any information on calculations on sample size or power. Future observational studies, should report their sample size calculation, i.e., reporting the assumed power to detect associations based on the sample size of the study population.

Third, accurate dietary assessment is known to be another methodological challenge. Most studies assessed dietary intake either a few days before milk sampling or by habitual intake using (multiple) 24-h dietary recalls, dietary records, weighted food records or food consumption records. Some studies used two dietary assessments methods (18, 20, 29, 32, 35–37, 41), and some others assessed dietary intake during pregnancy instead of lactation and used FFQs and dietary questionnaires that represent usual intake (14, 24, 28, 31, 32, 34). Studies scored higher points if they assessed diet during lactation, if the chosen assessment method was more appropriate, i.e., FFQ or at least three 24 h recalls or food records, and if the method was validated for the study population at hand. Judging the correct methods of dietary assessment however remains challenging, as little is known about the time needed for absorption and metabolize a nutrient, to eventually be present in human milk. Moreover, various studies focused on foods and food groups the exposure variable, which do not allow to identify whether the effect of a certain food (group) is due to its total composition or a specific nutrient, such as fish being rich in DHA.

Fourth, besides maternal dietary intake, several other factors have been related to human milk composition, which may impact the results and comparability of studies as well, such as: time postpartum (52), time of the day (53), variability within a feeding (54), and sampling method (55). Most included studies mention the postpartum period during which the samples were obtained, but several studies chose a very broad period and/or included milk samples from different lactational stages (transitional and mature) (Supplementary Table S3) (15, 16, 18, 26, 32, 38, 41, 43), or obtained samples at several timepoints, but did not specify which samples were used for association with dietary intake (20, 23, 39). Time postpartum is effecting all macro- and many micronutrients, with higher concentrations of (immune) proteins and lower concentrations of lactose in colostrum, compared to transitional and mature milk (52). Time postpartum at collection should therefore be as standardized across a study population as possible. Less is known on the exact nutritional composition over the day, yet especially amino acids are thought to be influenced by the circadian rhythm. During the day, levels of activity-promoting amino acids are higher compared to night time in mature milk (56). In addition, fat content in milk has been found to be higher during night time than during the day (53). Some studies did not specify the time of sampling (14, 16, 20, 21, 24, 26–30, 35, 43), but most studies collected milk at one time point during the day, often in the morning. Other studies obtained pooled human milk samples including morning, midday and evening feedings to limit the effect of circadian influences (15, 17, 18, 20, 25, 34, 41, 42). As mature human milk composition varies in composition of fore- to hindmilk, with an increasing fat content as the feeding proceeds (54), full expressed samples give the best representation of the human milk composition. Yet, only a few studies obtained full expressed human milk samples (16, 22, 30, 33, 35, 37, 38, 43), and most included studies obtained only fore- or only hindmilk samples (18, 19, 23, 32, 36, 39–41, 44), a pooled sample of fore- and hindmilk (17, 25, 34, 42) or did not provide information about milk type (Supplementary Tables S3, S4) (14, 20, 21, 24, 27–29, 31). To mitigate the possible effect of expression method, which possibly effect milk fat and protein concentration (57–59), 31% of all studies asked the participants to use a certain method for obtaining the milk samples, e.g., an electric pump or hand expressed. Studies scored higher points if they included samples within one lactational stage, standardized expression method and collected pooled samples of all feedings within a day and within a feeding.

As suggested by a recent systematic review, the composition of human milk samples may also be affected by storage and handling, such as pasteurization and various thawing and warming methods (60). Variations in storage and handling processes, make comparisons across studies challenging. Another methodological challenge across studies is the use of an appropriate method for analyzing the macro- and micro-nutrient content in human milk samples, as there is a wide array of different methods available (61). As an example, the two most commonly used methods to measure protein concentrations are the Dumas and bicinchoninic acid (BCA) methods. While Dumas indirectly quantifies protein content by measuring nitrogen levels and may therefore erroneously include non-protein nitrogen, the readings of the BCA technique are sensitive to false positives in case of insolubility or improper preparation of the sample and systematically overestimates protein content by up to 40% (62–64).

Lastly, many factors could play a confounding role in the association of maternal intake and human milk composition, such as maternal BMI, maternal age, breastfeeding status, and infant’s age. While most confounders can easily be studied, only six studies adjusted the observed associations for most known confounders (14, 16, 31, 34, 40, 43), and some studies adjusted for only part of the abovementioned confounding variables (18, 24, 30, 35, 37–40, 42, 44). Additionally, recent studies reported evidence for a difference in human milk composition between different races/ethnic groups for several compounds (65–67). However, among the included studies only a few showed the distribution of ethnic groups in the baseline characteristics (14, 19, 20, 24, 31, 33, 34), and only one study adjusted for ethnicity (14). As a consequence to all this variability in study set up and reporting, studies varied largely in their overall quality and comparability is limited.

## Conclusion

Since the last review on the association of maternal intake and human milk composition 6 years ago the body of evidence has increased significantly. Considering the total body of evidence to date, still most evidence is available for the association of maternal diet and human milk fatty acid composition. Among all fatty acids, PUFAs were studied most, mainly due to a large interest in DHA and EPA in human milk. Maternal DHA and fish intake were associated with higher human milk DHA level, confirming evidence from supplementation studies (68). While studied extensively, only few significant, but contradicting, associations were observed for maternal diet and human milk EPA concentration. Intake of carbohydrates, proteins, vitamins and minerals were nearly never significantly associated with human milk components. Overall, studies largely varied in their time postpartum, sampling techniques and overall quality, with 7 out of 35 studies scoring less than 50% of all possible quality indicators. Consequently, no strong conclusions can be drawn for most nutritional components. It becomes clear that research into the association of maternal intake and human milk composition is still diversified, with differences in study set up, outcome and exposure choice limiting the comparability of studies.

In order to give clear recommendations on the effects of maternal diet on human milk composition, more well-designed intervention or large observational studies according to a set of standardized pre-defined exposure and outcome variables are urgently needed.

## Author contributions

IP: Conceptualization, Investigation, Methodology, Validation, Visualization, Writing – original draft, Writing – review & editing. AH: Investigation, Validation, Visualization, Writing – original draft, Writing – review & editing. LL: Conceptualization, Funding acquisition, Supervision, Writing – review & editing. NK: Conceptualization, Investigation, Methodology, Writing – original draft. LB: Supervision, Writing – review & editing. KH: Supervision, Writing – review & editing. EF: Conceptualization, Funding acquisition, Methodology, Project administration, Resources, Supervision, Writing – review & editing. EB-B: Conceptualization, Methodology, Project administration, Resources, Supervision, Writing – original draft, Writing – review & editing.

## Footnotes

^1^ https://www.nhlbi.nih.gov/health-topics/assessing-cardiovascular-risk
