# Supplementary material for: Maternal diet and human milk composition: an updated systematic review
Source: Front Nutr. 2024 Jan 23;10:1320560. doi: 10.3389/fnut.2023.1320560 (PMC10862141; doi:10.3389/fnut.2023.1320560)
Supplement: Supplementary file 1 [file Table_1.DOCX]

**Appendix**

Table 1 Risk of bias assessment of included observational studies

|  | **Armand 2018** | **Aumeistere 2018** | **Aumeistere 2018** | **Aumeistere 2019** | **Azad 2018** | **Bobinski 2015** | **Butts 2018** | **Bzikowska-Jura 2018** | **Bzikowska-Jura 2019** | **Cai 2018** | **Daniels 2019** | **Deng 2018** | **Gibson 2020** | **Jagodic 2020** | **Jiang 2016** | **Jonsson 2016** | **Kim 2017** | **Kim, Yi 2017** | **Liu 2016** | **Liu 2019** | **Machado 2019** | **Miliku 2019** | **Perrin 2020** | **Williams 2016** | **Wong 2019** | **Zhang 2019** | **Zielinska 2019** |
| --- | --- | --- | --- | --- | --- | --- | --- | --- | --- | --- | --- | --- | --- | --- | --- | --- | --- | --- | --- | --- | --- | --- | --- | --- | --- | --- | --- |
| *Study population* |  |  |  |  |  |  |  |  |  |  |  |  |  |  |  |  |  |  |  |  |  |  |  |  |  |  |  |
| Sample size justification | 0 | 0 | 2 | 0 | 0 | 0 | 0 | 0 | 0 | 0 | 1 | 0 | 1 | 0 | 0 | 0 | 0 | 0 | 0 | 0 | 0 | 0 | 0 | 2 | 2 | 2 | 0 |
| Definition study population | 1 | 1 | 1 | 0 | 2 | 2 | 2 | 2 | 2 | 2 | 2 | 0 | 2 | 1 | 2 | 2 | 2 | 2 | 2 | 2 | 2 | 2 | 2 | 1 | 2 | 1 | 2 |
| *Exposure assessment* |  |  |  |  |  |  |  |  |  |  |  |  |  |  |  |  |  |  |  |  |  |  |  |  |  |  |  |
| Population characteristics | 1 | 1 | 1 | 1 | 2 | 1 | 1 | 2 | 2 | 1 | 2 | 0 | 2 | 1 | 1 | 1 | 2 | 2 | 2 | 1 | 1 | 2 | 1 | 1 | 1 | 1 | 1 |
| Time frame | 0 | 2 | 2 | 2 | 0 | 2 | 2 | 0 | 2 | 0 | 1 | 0 | 0 | 0 | 2 | 0 | 2 | 1 | 2 | 2 | 2 | 0 | 2 | 2 | 2 | 0 | 2 |
| *Exposure assessment* |  |  |  |  |  |  |  |  |  |  |  |  |  |  |  |  |  |  |  |  |  |  |  |  |  |  |  |
| Dietary assessment method | 2 | 2 | 2 | 2 | 2 | 2 | 2 | 1 | 2 | 2 | 2 | 0 | 2 | 2 | 0 | 1 | 2 | 2 | 2 | 2 | 2 | 2 | 2 | 2 | 2 | 2 | 1 |
| Validation of tools | 1 | 1 | 1 | 0 | 2 | 0 | NA | 0 | NA | 0 | 0 | 0 | NA | 0 | 0 | 1 | NA | NA | 0 | 1 | 0 | 0 | 0 | 0 | NA | 1 | 2 |
| *Outcome assessment* |  |  |  |  |  |  |  |  |  |  |  |  |  |  |  |  |  |  |  |  |  |  |  |  |  |  |  |
| Adjustment for confounders | 1 | 0 | 0 | 1 | 2 | 0 | 0 | 0 | 0 | 0 | 1 | 0 | 1 | 0 | 1 | 0 | 2 | 2 | 2 | 0 | 0 | 2 | 1 | 2 | 1 | 1 | 1 |
| *Milk sampling* |  |  |  |  |  |  |  |  |  |  |  |  |  |  |  |  |  |  |  |  |  |  |  |  |  |  |  |
| Specificity of time postpartum | 2 | 1 | 1 | 1 | 2 | 2 | 2 | 1 | 0 | 0 | 2 | 0 | 2 | 2 | 0 | 2 | 1 | 0 | 2 | 0 | 2 | 2 | 1 | 2 | 0 | 2 | 2 |
| Expression method | 2 | 0 | 0 | 0 | 1 | 0 | 0 | 0 | 0 | 0 | 0 | 0 | 2 | 0 | 0 | 2 | 2 | 2 | 0 | 2 | 2 | 0 | 0 | 2 | 0 | 0 | 0 |
| Type of milk sample | 0 | 1 | 1 | 1 | 2 | 0 | 1 | 2 | 2 | 0 | 1 | 0 | 1 | 1 | 0 | 0 | 1 | 1 | 0 | 0 | 0 | 1 | 1 | 0 | 1 | 0 | 2 |
| *Other* |  |  |  |  |  |  |  |  |  |  |  |  |  |  |  |  |  |  |  |  |  |  |  |  |  |  |  |
| Funding of the study | 1 | 2 | 2 | 2 | 2 | 0 | 0 | 2 | 2 | 0 | 2 | 0 | 2 | 2 | 2 | 2 | 1 | 1 | 0 | 2 | 2 | 2 | 2 | 2 | 2 | 0 | 2 |
| *Total (max. 22 or 20*)* | **11** | **11** | **13** | **10** | **17** | **9** | **10*** | **10** | **12** | **5*** | **14** | **0** | **15** | **9*** | **8** | **11** | **15*** | **13*** | **12** | **12** | **13** | **13** | **12** | **16** | **13** | **10*** | **15** |
| **% of total** | **50** | **50** | **59** | **45** | **77** | **41** | **50** | **45** | **60** | **23** | **64** | **0** | **75** | **41** | **36** | **50** | **75** | **65** | **55** | **55** | **59** | **59** | **55** | **73** | **65** | **45** | **68** |

Table 2 Risk of bias assessment of included intervention studies

|  | **Berger et al., 2018** | **Jochum et al., 2017** | **Metcalfe et al., 2016** | **Ward et al., 2021** | **Yahvah et al., 2015** |
| --- | --- | --- | --- | --- | --- |
| ***Blinding and randomization*** |  |  |  |  |  |
| Randomization | 2 | 2 | 1 | 0 | 0 |
| Concealed treatment allocation | 0 | 0 | 0 | 0 | 0 |
| Blinding | 0 | 0 | 1 | 0 | 0 |
| ***Study population*** |  |  |  |  |  |
| Similarity at baseline | 2 | 1 | 1 | 2 | 2 |
| Sample size justification | 2 | 0 | 2 | 0 | 0 |
| ***Follow-up*** |  |  |  |  |  |
| Drop out | 2 | 2 | 1 | 2 | 2 |
| ***Outcome assessment*** |  |  |  |  |  |
| Analysed as prespecified | 0 | 2 | 2 | 2 | 2 |
| ***Milk sampling*** |  |  |  |  |  |
| Specificity of time postpartum | 2 | 0 | 2 | 2 | 2 |
| Expression method | 2 | 2 | 0 | 0 | 2 |
| Type of milk sample | 2 | 0 | 0 | 1 | 1 |
| ***Other*** |  |  |  |  |  |
| Funding of the study | 2 | 0 | 1 | 2 | 0 |
| ***Total (max. 22)*** | ***16*** | ***9*** | ***11*** | ***11*** | ***11*** |
| **%** | **73** | **41** | **50** | **50** | **50** |

*Table 3 Overview observational studies*

| **Ref** | **Author (year)** | **Country** | **Study design** | **Participants** (n, age±SD) | **Milk sampling procedure**  (time postpartum, expression method, storage, time of day, etc.) | **Type of milk** | **Exposure type** |
| --- | --- | --- | --- | --- | --- | --- | --- |
| [23] | Armand et al., 2018 | France | cross-sectional (sub group of prospective cohort) | n=803, mean age 29.6±4.7 | 2-5 days postpartum (5mL), manual expression | colostrum | Nutrient level |
| [34] | Azad et al., 2018 | Canada | cross-sectional (sub group of prospective cohort) | n=427 mean age 33.0±4.2y | 3-4 months postpartum, fore- and hindmilk of several feedings during 24h, hand expression encouraged | mature | Nutrient level |
| [16] | Aumeistere et al., 2019 | Latvia | cross-sectional | n=61,  median age 31 (range 23-39) | >1 month postpartum, pooled hindmilk sample over 24h (morning, midday, evening) (60mL), free to choose expression method | mature | Nutrient level |
| [40] | Aumeistere et al., 2018 | Latvia | cross-sectional | n=62 mean age 31±4y | >1 month postpartum, pooled hindmilk sample over 24h (morning, midday, evening) (10mL), free to choose expression method | mature | Nutrient level |
| [32] | Aumeistere, L. et al., . 2018 | Latvia | cross-sectional | n=67, mean age 31±4 | >1 month postpartum, pooled hindmilk sample over 24h (morning, midday, evening) (10mL), free to choose expression method | mature | Food product level |
| [28] | Bobiński at el., 2015 | Poland | cross-sectional | n=95 mean age 28.6±4.7 | 4-7days postpartum, 10mL | transitional | Nutrient level |
| [18] | Butts et al., 2018 | New Zealand | cross-sectional | n= 78 mean age 31±5 | 6-8 weeks postpartum, 3 samples expressed over 1 week (50mL each) from first feed of the day, expressed by hand or pump | mature | Nutrient level |
| [15] | Bzikowska-Jura et al., 2018 | Poland | longitudinal | n=40 mean age 31.1±4.4 | 1st, 3rd, 6th month of lactation. Pooled pre-feed a post-feed milk samples (5-10mL) over 24h (fixed times). Manually expressed or by pump | mature | Nutrient level |
| [24] | Bzikowska-Jura et al., 2019 | Poland | cross-sectional | n=32 mean age 30.9±6.5 | 2-4 weeks postpartum. Pooled fore- and hindmilk samples (5-10mL) over 24h (fixed times) | mature | Nutrient level |
| [19] | Cai et al., 2018 | China | cross-sectional | n=248 age 20-35 | 17–30 days, 31–90 days, 91–150 days, 151–240 days and 241–330 days postpartum, kept frozen at -80 | mature | Food group level |
| [35] | Daniels et al., 2019 | Indonesia | cross-sectional | n=113 mean age 25.8 ± 6.1 | 2-5.5 months postpartum, full expression either manually or via pump (1mL) in the morning | mature | Nutrient level |
| [25] | Deng et al., 2018 | China | cross-sectional | n=65 age 22-41 | - | colostrum & mature | Nutrient level |
| [37] | Gibson et al., 2020 | Indonesia | longitudinal | n=193 mean age 28.4±6.4 | 2nd and 5th month of lactation, full expression | mature | Nutrient level |
| [26] | Jagodic, et al., 2020 | Slovenia | cross-sectional | n=74 mean age 29 | 5-11 weeks postpartum, pooled sample of multiple feeds within 6 days | mature | Food group level |
| [38] | Jiang, Xiao, et al., 2016 | China | longitudinal | n=102 mean age 28.13 | 1, 14 and 42 days postpartum, foremilk of morning feed (10 am -11 am) (35mL), collected in dim lit room and into light protected containers | colostrum, transitional and mature | Food group level |
| [29] | Jonsson et al., 2016 | Sweden | cross-sectional | n=65 mean age 32.5 | 4 months postpartum, manual expression (5mL) at any time of the day | mature | Food group level |
| [14] | Kim et al., 2017 | South Korea | cross-sectional | n=238 mean age 31.6 | Full expression by pump | not specified | Nutrient level |
| [43] | Kim, Yi, et al., 2018 | South Korea | cross-sectional | n=98 mean age 32.5±3.5 | Full expression by pump | not specified | Nutrient level |
| [22] | Liu, et al., 2019 | China | cross-sectional | n=2007 mean age 27.9 | 1-7 days and 42 postpartum. Foremilk (first drops were discarded). Manual expression (10mL) during morning feed | colostrum, transitional and mature | Nutrient level |
| [31] | M. J. Liu et al., 2016 | China | cross-sectional | n=408 mean age 27.34±2.97 | 42 days postpartum. Manual or pump expression (10mL) during morning feed | mature | Nutrient level |
| [36] | Machado et al., 2019 | Brazil | longitudinal | n=19  mean age 31.9±5.9 | 2nd-4th and 12th-14th week postpartum, manual expression of 10mL in the morning | transitional, mature | Nutrient level |
| [27] | Miliku et al., 2019 | Canada | cross-sectional | n= 1094 mean age 32.9±4.3 | 3-4 months postpartum, 10mL collected over 2 or more feedings. Manual and pump expression (although hand expression preferred). Kept frozen at -80 | mature | Diet |
| [41] | Perrin et al., 2020 | USA | cross-sectional | n=74 | >2 weeks postpartum, full expression, collected in the morning during 1st or 2nd feed of the day. | mature | Diet |
| [39] | Williams et al., 2016 | Kenya | cross-sectional | n=286 mean age 25 | 1-6 months postpartum, 5mL collected 1min into feeding by manual expression, collected in the morning (9-12h) | mature | Nutrient level |
| [30] | Wong et al., 2019 | Hong Kong | cross-sectional | n= 73 mean age 32.1 ±3.7 | full expression by pump or manual | mature | Food product level |
| [44] | Zhang et al., 2019 | China | longitudinal | n=37 mean age 28.92±4.27 | 4th, 8th and 12th month postpartum 30mL sampled in the morning, limited exposure to light and oxygen | mature | Nutrient level |
| [42] | Zielinska, et al., 2019 | Poland | longitudinal | n=49 mean age 31.4±3.8 | 3rd and 6th month postpartum, fore- and hindmilk (5-10mL) collected over 24h. Manually or by pump | mature | Nutrient level |

Table 4 Overview trials

| **Ref** | **Author (year)** | **Country** | **Study design** | **Participants** (n, age±SD) | **Milk sampling procedure**  (time postpartum, expression method, storage, time of day, etc.) | **Type of milk** | **Exposure type** |
| --- | --- | --- | --- | --- | --- | --- | --- |
| [33] | Berger et al., 2018 | USA | crossover trial, 6h intervention time, 3 day wash out period | n=41 mean age 29.8±2.9 | 6 weeks postpartum, complete expression over 6h by pump | mature | High-fructose corn-syrup-sweetened beverage vs. no caloric sugar/artificially sweetened control beverage |
| [45] | Jochum et al., 2017 | Germany | RCT, 6 days intervention | n=44 mean age 30.3 | 15-27 days postpartum before intervention, during intervention days and after (>10mL), 5min into feeding during evening by breast pump | mature | Black tea vs. soy milk |
| [20] | Metcalfe et al., 2016 | Australia | RCT, 6 weeks intervention | n=120 mean age 33 | 2,4 and 6 weeks postpartum (10mL) | transitional and mature | High egg vs. low egg vs. egg-free diets |
| [17] | Ward et al., 2021 | Australia | crossover trial, 1 day intervention, 1 week wash out period between 3 intervention groups | n=10 mean age 33.6 | 6-24 weeks, 24h collection by pump or manual (> 2mL) | mature | High fat vs. high sugar vs. control |
| [21] | Yahvah et al., 2015 | USA | crossover trial, 14 days intervention, 2 weeks wash out period | n=15 mean age 27±1 | full expression by pump between 6.00 and 10.00 | mature | Full fat vs. low fat dairy diet |
